# Book Notices

**Published:** 1886-06-20

**Authors:** 


*Diseases of the Urinary and Male Sexual Organs.* By William T. Belfield, M. D., author of "Relations of Micro-Organisms to Disease;" Pathologist to the Cook County Hospital; Surgeon to the Genito-Urinary Department Central Dispensary, Chicago, etc , etc. William Wood & Co., Publishers, New York.

Among the many points worthy of mention in this book, we note the remark of the author that the kidneys being a part of the urinary tract, that there is danger of hyperæmia and inflammation of this organ from sudden removal of obstructions in cases of stricture or other causes of interruption to the full evacuation of the bladder. This is a very important practical point, and is strongly insisted on in many places throughout the work. Knowing, and duly estimating the danger from this source, the inference as to practice is plain: *never empty the bladder fully in any case when there is residual urine, or incomplete evacuation of this organ.*

The prevalence of enuresis or incontinence of urine in childhood is satisfactorily accounted for on anatomical and physiological principles. The sphincters of the bladder being two, the internal or prostatic and the external, or urethral surrounding the membranous urethra. He explains incontinence in early life thus: "Previous to puberty, it appears to be the result simply of a lack of development in the internal sphincter, for this sphincter is a part of the prostate, and this gland, in common wi:h the genital organs generally, remains undeveloped during the first years of life. The compress or urethal, surrounding t'ie membranous urethra, which is a striped muscle, and under voluntary control, suffices for the closing of the bladder during the waking hours; but in sleep this control is less perfectly exercised, and because it is not reinforced by the internal sphincter, nocturnal incontinence often results." On the same principle enuresis of old men is accounted for; the muscular fibres of the bladder and prostate having undergone fatty degeneration. When thhe detrusor muscles are affected, ability to expel the urine is impaired, and there is over distension, paralysis of the bladder, etc.

For exploration of the urethra our author recommends bullous sounds, and the Otis urethometes, to the exclusion of conical instruments.

The position recommended for the introduction of catheters, etc., is the recumbent. His remarks upon the causes of *urethral fever;* upon digital exploration of the bladder in women; upon the use of the endscope, the clinical examination of the urine; seminal incontinence, etc., are interesting and instructive.

